# Prenatal Ultrasound Diagnosis of Congenital Diaphragmatic Hernia in a Fetus With Fryns “Anophthalmia-Plus” Syndrome: A Case Report

**DOI:** 10.7759/cureus.68000

**Published:** 2024-08-28

**Authors:** Vasiliki-Areti Maritsa, Alexandros Psarris, Antonios Koutras, Paraskevas Perros, Zacharias Fasoulakis, Andreas Pampanos, Panagiotis Antsaklis, Michael Sindos, George Daskalakis, Marianna Theodora

**Affiliations:** 1 1st Department of Obstetrics and Gynecology, Alexandra Maternity Hospital, National and Kapodistrian University of Athens, Athens, GRC; 2 Department of Genetics, National and Kapodistrian University of Athens, Athens, GRC; 3 1st Department of Obstetrics and Gynecology, National and Kapodistrian University of Athens, Athens, GRC; 4 1st Department of Obstetrics and Gynecology, Alexandra General Hospital, National and Kapodistrian University of Athens, Athens, GRC

**Keywords:** anophthalmia, pulmonary hypoplasia, distal limp hypoplasia, craniofacial anomalies, congenital diaphragmatic hernia, fryns syndrome

## Abstract

Fryns syndrome is an extremely rare autosomal recessive disorder and is characterized by congenital diaphragmatic hernia (CDH), dysmorphic facial features, distal limb hypoplasia, pulmonary hypoplasia, and characteristic-associated anomalies that lead to a high mortality rate. We present a prenatally diagnosed new case of Fryns “anophthalmia-plus” syndrome (FAPS) in a 41-year-old pregnant woman. An ultrasonographic examination at 22 weeks of gestation demonstrated left CDH with mediastinal shift, hypoplastic thorax with presumptive pulmonary hypoplasia, craniofacial anomalies, left anophthalmia, and distal limb hypoplasia. A genetic analysis of the fetal karyotype was held, which was negative for any known chromosomal or single gene abnormalities. After genetic counseling about the risks associated with these ultrasonographic findings, the parents opted for pregnancy termination. Timely identification or suspicion of Fryns syndrome during the early stages of pregnancy could facilitate parental guidance and enable the development of suitable strategies for prenatal treatment and/or perinatal care.

## Introduction

Fryns syndrome is a rare disorder that is believed to comply with the rules of autosomal recessive inheritance pattern. The prevalence in the general population is estimated to be 0,7 per 10000 births [1]. The syndrome was documented for the first time by Fryns in 1979 [2]. The incidence in patients with congenital diaphragmatic hernia (CDH) has been estimated to be 1,3% [3]. The incidence of the syndrome can vary significantly, ranging from 4% to 10%. This variation is due to the utilization of different methods to confirm the presence of the syndrome, the growing number of cytogenetic and molecular techniques used to identify or rule out the syndrome, and the increasing accessibility of accurate prenatal diagnosis.

Fryns syndrome is characterized by the presence of CDH of any location in 90% of the cases, craniofacial anomalies (Dandy-Walker Syndrome, agenesis of the corpus callosum, orofacial clefting, coarse and flat face combined with microphthalmia and hypertelorism, flat nasal bridge, micrognathia, facial hair overgrowth, and low-set, poorly formed ears) and distal digital hypoplasia that is usually combined with absent or hypoplastic finger nails and short terminal phalanges. Furthermore, pulmonary hypoplasia with hypoplastic thorax and wide-distanced nipples and characteristic-associated anomalies, such as renal cortical cysts, renal dysplasia, polyhydramnios, genital malformations, cardiovascular malformations, and gastrointestinal malformations, may be observed in 86% of the cases [4,5].

Survival after the newborn stage is uncommon. There is a lack of information regarding postnatal growth and psychomotor development. However, it is frequently observed that individuals experience significant developmental delay and intellectual disability [6]. We present a case of prenatal ultrasound diagnosis of Fryns syndrome in a female fetus.

## Case presentation

**Figure 1:**
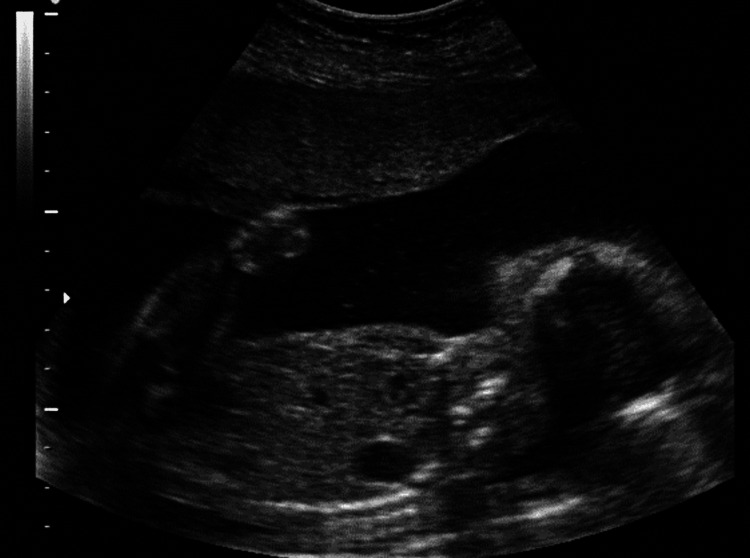
Left diaphragmatic hernia with localization of the stomach into the thoracic cavity

A 41-year-old patient (gravida 2, para 0), during the 22nd week of gestation, was referred for a regular level II ultrasound fetal anatomy scan. From the medical history, we gathered the information that the patient was suffering from an autosomal recessive malabsorption syndrome, called abetalipoproteinemia (Bassen-Kornzweig syndrome), where large doses of fat-soluble vitamins are needed. It is caused by a mutation in microsomal triglyceride transfer protein, resulting in deficiencies in the apolipoproteins B-48 and B-100, which are used in the synthesis and exportation of chylomicrons and very-low-density lipoprotein (VLDL), respectively [7]. The treatment involved the administration of vitamins A, D, E, and K, together with polyethylene glycol. The patient exhibited a low body mass index (BMI = 17.6) and a thin figure. The patient disclosed a first-trimester abortion in a previous pregnancy and experienced modest bleeding in the first trimester of the current pregnancy. The ultrasound scan revealed the existence of a unilateral left-sided CDH with mediastinal shift and hypoplastic thorax (Figure 1).

**Figure 2:**
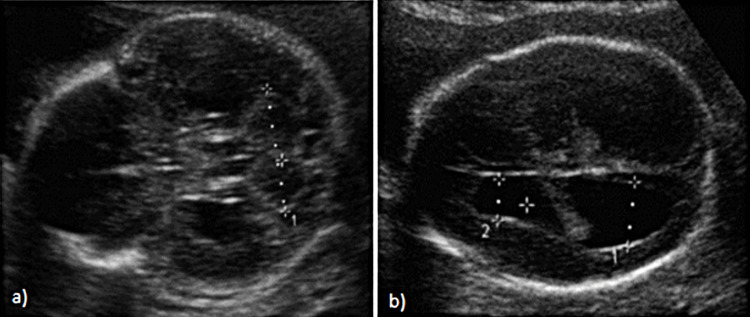
(A) Fetal "cloverlike" skull. (B) Anterior and posterior mild ventriculomegaly with choroid plexus displacement (D1 = 15 mm; D2 = 9.58 mm)

The hernia’s content consisted exclusively of the stomach. Multiple craniofacial anomalies were noted such as “cloverlike” cranium (Figure 2A), mild bilateral ventriculomegaly (Figure 2B), hypoplastic nasal bone (4 mm), low-set ears, hypertelorism, and micrognathia. Significant right microphthalmia and left anophthalmia with absent imaging of the left cornea and protuberance of the frontal bone were observed, resulting in a flat face.

The three-dimensional (3D) imaging of the fetus indicated a coarse face. Distal digital hypoplasia was indicated by the measurements of all long bones. The nuchal fold was estimated at 6,5 mm. The amniotic fluid index and the umbilical cord vessels were normal. No other fetal abnormalities were revealed.

**Figure 3:**
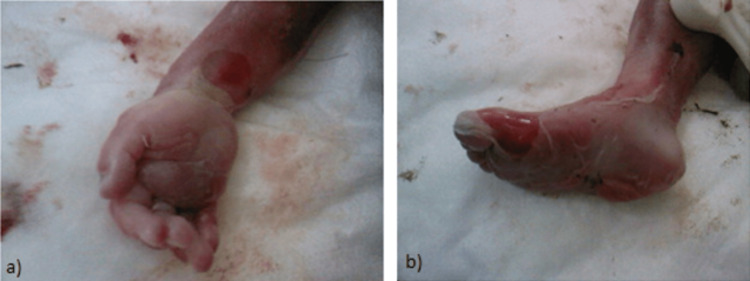
(A-B) Postmortem photos showing edema and short-end phalanges of the distal limbs

**Figure 4:**
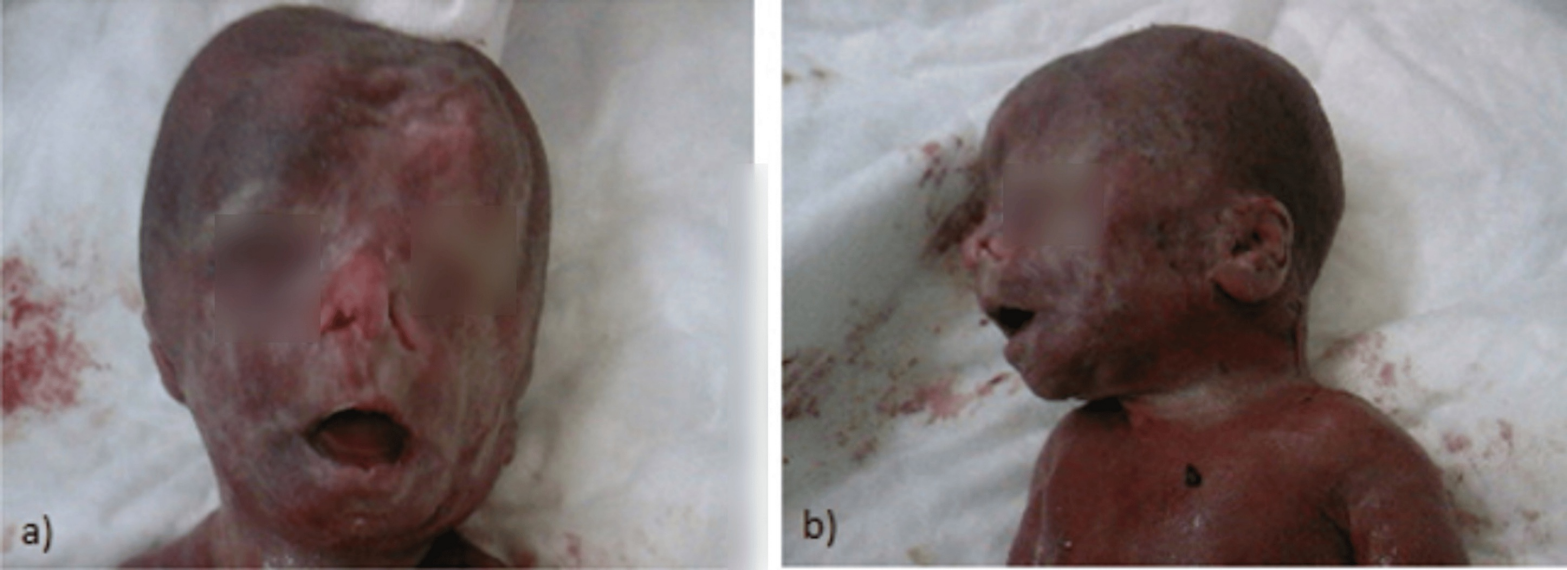
(A) The left anophthalmia, right microphthalmia, left nasal cleft, and coarse face. (B) The flat face, micrognathia, and low-set malformed ears

As described above, the ultrasound detected multiple structural defects. Those findings suggested the existence of a genetic syndrome or chromosomal abnormality. An analysis of the fetal karyotype was recommended, an amniocentesis was held and revealed normal female karyotype. After genetic counseling about the risks associated with these ultrasonographic findings, parents have decided to proceed to fetal abortion and were referred to the outpatient clinic of our hospital for further management of the pregnancy. Postmortem observation after delivery established that the fetus had hypoplasia and edema of distal limbs (Figures 3A, 3B) and left anophthalmia and right microphthalmia (Figure 4A). The left diaphragmatic hernia and pulmonary hypoplasia remained an ultrasound finding, as parents did not consent to perform autopsy or MRI scanning. The craniofacial anomalies that were detected by the ultrasound scan were confirmed, while a left nasal cleft was also noted (Figures 4A, 4B).

The fetal blood sample was received to verify the karyotype result. The result was negative for any known genetic abnormalities. These findings strongly suggest a fetus with Fryns “anophthalmia-plus” syndrome (FAPS).

## Discussion

Fryns syndrome is a rare autosomal recessive disorder with a high probability of causing stillbirths or neonatal deaths. No specific pathogenic gene defect is associated yet with the syndrome. Significant intra- and interfamily phenotypic variability, as well as discordant phenotype in monozygotic twins, has been observed [5]. However, certain described chromosomal abnormalities sometimes accompany the phenotype of the syndrome, and these include the accidental mosaic duplication of region 1q24-q31.2, the final deletion of region 6q, and trisomy 22, while recent publication raises the question of possible correlation of Fryns and Turner syndrome (45XO) [8-11].

The syndrome is believed to have a higher risk when siblings are affected and/or when there is consanguinity between parents [12,13]. The concept that FAPS is a unique disorder is supported by the findings in several published cases [8,14]. The genetic location of FAPS has not yet been determined. The prognosis of the disease is bleak since the majority of the fetuses are either stillborn or perish during the early neonatal stage of life due to significant underdevelopment of the lungs. All neonates who have survived without diaphragmatic hernia have exhibited severe mental impairment in rare instances [15,16].

Fryns syndrome can be clinically diagnosed in an individual by assessing six specific criteria: diaphragmatic defect, characteristic facial appearance, distal digital hypoplasia, pulmonary hypoplasia, presence of at least one associated anomaly, and a family history that aligns with autosomal recessive inheritance. Prenatal recognition of CDH, using routine two-dimensional (2D) or 3D ultrasound, is necessary and is the only way to suspect Fryns syndrome and determine which embryos can survive. The most typical and frequent findings are hypoplasia of the limbs, malformed and low-set ears, deformed facial characteristics, and hypoplastic thorax. The presence of diaphragmatic hernia is the prevalent diagnostic criterion for Fryns syndrome, while pulmonary hypoplasia is the main mortality cause. Pregnancies should be managed according to the malformations that have been diagnosed and after the appropriate genetic counseling. Molecular diagnosis can be confirmed in an individual with suggestive symptoms and two pathogenic mutations in the phosphatidylinositol glycan anchor biosynthesis class N (PIGN) gene found using molecular genetic testing [6].

The syndrome’s exclusion criteria are very important to be formed, as many chromosome aberrations have been associated with CDH and major malformations. The method of microarray-DNA karyotyping should also be used to verify or exclude the syndrome’s existence [17,18]. Specifically, appropriate control should be performed to exclude the following chromosome aberrations that have been associated with CDH and additional malformations: isochromosome 12p (mosaic tetrasomy 12p, Pallister-Killian syndrome), partial trisomy for chromosome 22q, deletion of chromosomes 15q26.2, 8p23.1, 1q41-1q42, and de novo copy number variants [19-25]. Single-gene disorders in which CDH can be observed are Simpson-Golabi-Behmel syndrome, Cornelia de Lange syndrome, Donnai-Barrow syndrome, and Matthew-Wood syndrome [26-31].

In cases of CDH, the newborn is promptly intubated to avoid the expansion of the herniated bowel. This is followed by surgical intervention and/or supportive treatments, similar to those used for the general population. Antiepileptic drugs were administered according to established protocols by a skilled neurologist. Further abnormalities may necessitate consultations and treatment by specialists in ophthalmology, cardiology, gastrointestinal, nephrology, urology, and craniofacial medicine. Necessary developmental services, such as nutrition, motor, adaptive, cognitive, and speech/language therapy, should also be provided. Individuals who have undergone successful surgery of CDH should get ongoing monitoring at a specialized center. This monitoring should include regular evaluations by a multidisciplinary team consisting of a pediatric surgeon, nurse specialist, cardiologist, pulmonologist, and nutritionist [6,32].

## Conclusions

Fryns syndrome is an uncommon genetic disorder that is inherited in an autosomal recessive manner. It has a significant likelihood of resulting in stillbirths or deaths of newborns. This highlights the importance of early identification in subsequent pregnancies. Pregnancies should be managed according to the malformations that have been diagnosed and after the appropriate genetic counseling. Parents must also be notified about the likelihood of a repeated occurrence in another pregnancy. Unfortunately, no specific pathogenic gene defect is associated yet with the syndrome, and future research should focus on revealing the gene locus of the syndrome. It is crucial to differentiate it from other syndromes that exhibit similar symptoms but encompass many defects.
